# Malaria-VisAnalytics: a tool for visual exploratory analysis of Brazilian public malaria data

**DOI:** 10.1186/s12936-022-04248-w

**Published:** 2022-08-01

**Authors:** Alberto Pietro Sironi, Juracy Bertoldo, Vanderson Sampaio, Danilo Coimbra, Davide Rasella, Marcos Ennes Barreto

**Affiliations:** 1 AtyImoLab – Institute of Computing, Federal University of Bahia (UFBA), Salvador, Brazil; 2 Amazonas State Foundation for Health Surveillance (FVS-AM), Manaus, Brazil; 3 Institute of Collective Health, Federal University of Bahia (UFBA), Salvador, Brazil; 4 Department of Statistics, London School of Economics and Political Science (LSE), London, UK

**Keywords:** Data mining, Data linkage, Spatial and non-spatial data visualisation, Visual knowledge discovery

## Abstract

**Background:**

Data integration and visualisation techniques have been widely used in scientific research to allow the exploitation of large volumes of data and support highly complex or long-lasting research questions. Integration allows data from different sources to be aggregated into a single database comprising variables of interest for different types of studies. Visualisation allows large and complex data sets to be manipulated and interpreted in a more intuitive way.

**Methods:**

Integration and visualisation techniques were applied in a malaria surveillance ecosystem to build an integrated database comprising notifications, deaths, vector control and climate data. This database is accessed through Malaria-VisAnalytics, a visual mining platform for descriptive and predictive analysis supporting decision and policy-making by governmental and health agents.

**Results:**

Experimental and validation results have proved that the visual exploration and interaction mechanisms allow effective surveillance for rapid action in suspected outbreaks, as well as support a set of different research questions over integrated malaria electronic health records.

**Conclusion:**

The integrated database and the visual mining platform (Malaria-VisAnalytics) allow different types of users to explore malaria-related data in a user-friendly interface. Summary data and key insights can be obtained through different techniques and dimensions. The case study on Manaus can serve as a reference for future replication in other municipalities. Finally, both the database and the visual mining platform can be extended with new data sources and functionalities to accommodate more complex scenarios (such as real-time data capture and analysis).

## Background

Data visualisation and exploratory analysis techniques have been used for decades in scientific research to support data understanding and possibly providing evidence for generating new hypothesis to be tested. Particularly, for analysing high dimensional data, an inherent challenge to data management after data pre-processing is displaying results in an intuitive way. Many data visualisation tools have emerged in the recent years, aiming to support visual processing and mining of large amounts of data, contributing significantly to adequate decision-making processes [1].

Additionally, in recent years, there has been an increasing availability of data from monitoring systems, particularly health systems (such as mortality, morbidity, and hospitalizations), that can take advantage of data visualisation techniques to evaluate trends of health outcomes, associative effects of exposures and outcomes, and impact of public policies. Especially for malaria, the literature comprises proposals of Web applications and different data visualisation techniques providing support for querying, visualisation, and execution of spatio-temporal analysis of malaria data.

Visualisation based on global maps allows users to easily perceive the global extent of malaria. The Malaria Atlas project [2] is a cooperative work between the World Health Organization (WHO) and partnering institutions to develop a set of interactive maps to quantify malaria syndromes and treatment rates. The Web platform provides the generation of a global map of dominant vector species of malaria that makes use of predicted distribution for individual species. The global map application highlights the spatial variability in the complexity of the vector situation. The proposed tool has a similar functionality, showing the percentage of cases of malaria and the respective dominant species for each region.

A similar work was presented in Visualize-No-Malaria [3]. The platform proposes a real time analysis to promptly identify and treat every new case of malaria. It is targeted to settings where the parasite is hidden and is built upon accurate and reliable data tracking emerging transmission patterns. Using the platform, it is possible to visualize and monitor insecticide-treated bed nets, indoor residual spray, rapid malaria diagnostic tests and drugs.

A system for viewing and monitoring malaria cases in Brazil was described by Prettz et al. [4], who showed the importance of monitoring and establishing new policies for disease prevention. Different graphs were generated with the aim of elucidating pertinent information of malaria. The results indicate that the greater part of changes in the notifications occur in regions of the Amazon Forest, and that the majority of those infected are male.

Network visualisation is a powerful approach to discover relationships among different data. A platform allowing researchers to easily explore, analyse, and compare graph data in an interactive way by selecting (or filtering) data points (representing graphs, nodes, and edges) across a variety of important and fundamental graph statistics and properties is presented by Rossi et al. [5]. Intuitively, this filtering and selection tool highlights all nodes that have certain properties of interest, such as those that have a triangle count in certain user-defined range. This interactive platform gives rise to an infinite number of ways to visualize and compare data in a real time.

A spatial auto-correlation analysis showing the geographic association of districts with malaria outbreaks and their direct or indirect association with reported cases in neighbouring districts is discussed by Zhou et al. [6], who suggested a putative relation between regional similarities in climate and environmental factors and the dynamics of transmission vectors. High spatial variation in malaria incidence and local human migration patterns suggest that malaria control measures need to be adjusted according to local environmental and demographic settings.

This work takes a closer look at the distribution of malaria in Brazil and its associated factors. Malaria remains a worldwide public health problem. In Brazil, there are high rates of incidence, especially in the Amazonian region, where environmental conditions promote the proliferation of mosquitoes [7]. The Brazilian malaria surveillance system records malaria episodes into two different databases managed by the Ministry of Health: Malaria Epidemiological Surveillance System (SIVEP) and Notifiable Diseases Information System (SINAN).

In this work, data were aggregated from these systems, as well mortality, climate, and vector control data, into a R-Shiny Web-based platform providing interactive metaphors for visual analysis and pattern identification. The proposed tool helps to improve the mapping and understanding of malaria distribution in Brazil, as well as its relationship with environmental and socioeconomic factors, in a simple and intuitive way.

## Methods

**Fig. 1:**
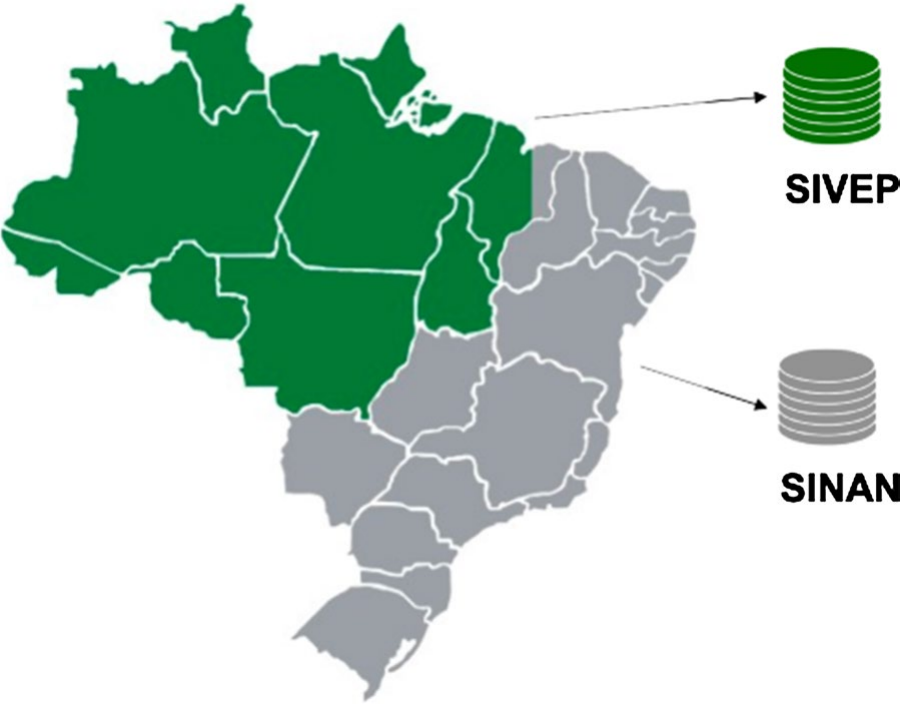
Brazilian databases recording malaria episodes within (SIVEP—epidemiological surveillance system (green)) and outside (SINAN—notifiable diseases system (grey)) the Amazonian region

The Brazilian malaria surveillance system comprises databases capturing data inside and outside the Brazilian Amazonian region. These databases are, respectively, SIVEP and SINAN, as depicted in Fig. 1.

SIVEP is an information system storing data about malaria cases reported in the health units and medical offices inside the Brazilian Amazonian region [8]. The database covers data from 2003 to 2017 with 5,490,603 records and 43 attributes. SINAN is epidemiological surveillance system recording cases of more than twenty contagious diseases, including malaria, outside the Amazonian region [7]. The database covers the period 2003 to 2018 with 42,670 records and 79 attributes.

These databases are supplemented with municipal-level data from the mortality information system (SIM), a system of regular registration of mortality data in the country. Climate data, obtained from the National Oceanic and Atmospheric Administration [9], were also integrated into the platform. These data cover daily temperature, humidity, precipitation and ground temperature for all the 5,570 Brazilian municipalities for the period 2003 to 2018.

The linkage process has been performed in two steps: first, the individual-level datasets (SIVEP, SINAN) have been collapsed to become aggregate panel datasets at the municipal level. Municipal panel datasets have repeated (yearly) information for each municipality and for each attribute. Second, these municipal-level panel datasets have been linked through a deterministic method with mortality (SIM) and climate data (already panel data at the municipal level). The deterministic linkage was based on two unique exact identifiers: the municipal code (composed of 6 numbers) from the Brazilian Institute of Geography and Statistics (IBGE) and the year of the observations. Such deterministic linkage does not allow any false match and is not prone to typical errors of probabilistic linkage; for these reasons it is a consolidated method used in studies that integrates information from different data sources in Brazil [10, 11].

**Fig. 2:**
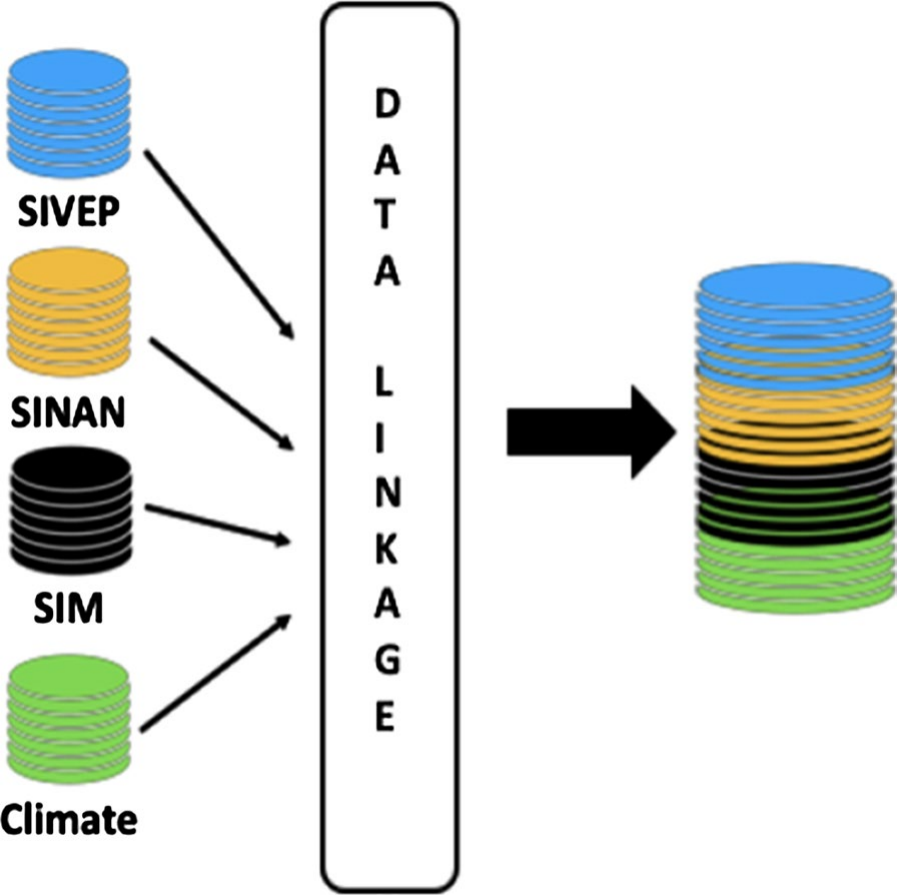
Data sources linked to build a national database of malaria episodes and related data: SIVEP (epidemiological surveillance system), SINAN (notifiable diseases system), SIM (death records), and climate data

**Fig. 3:**
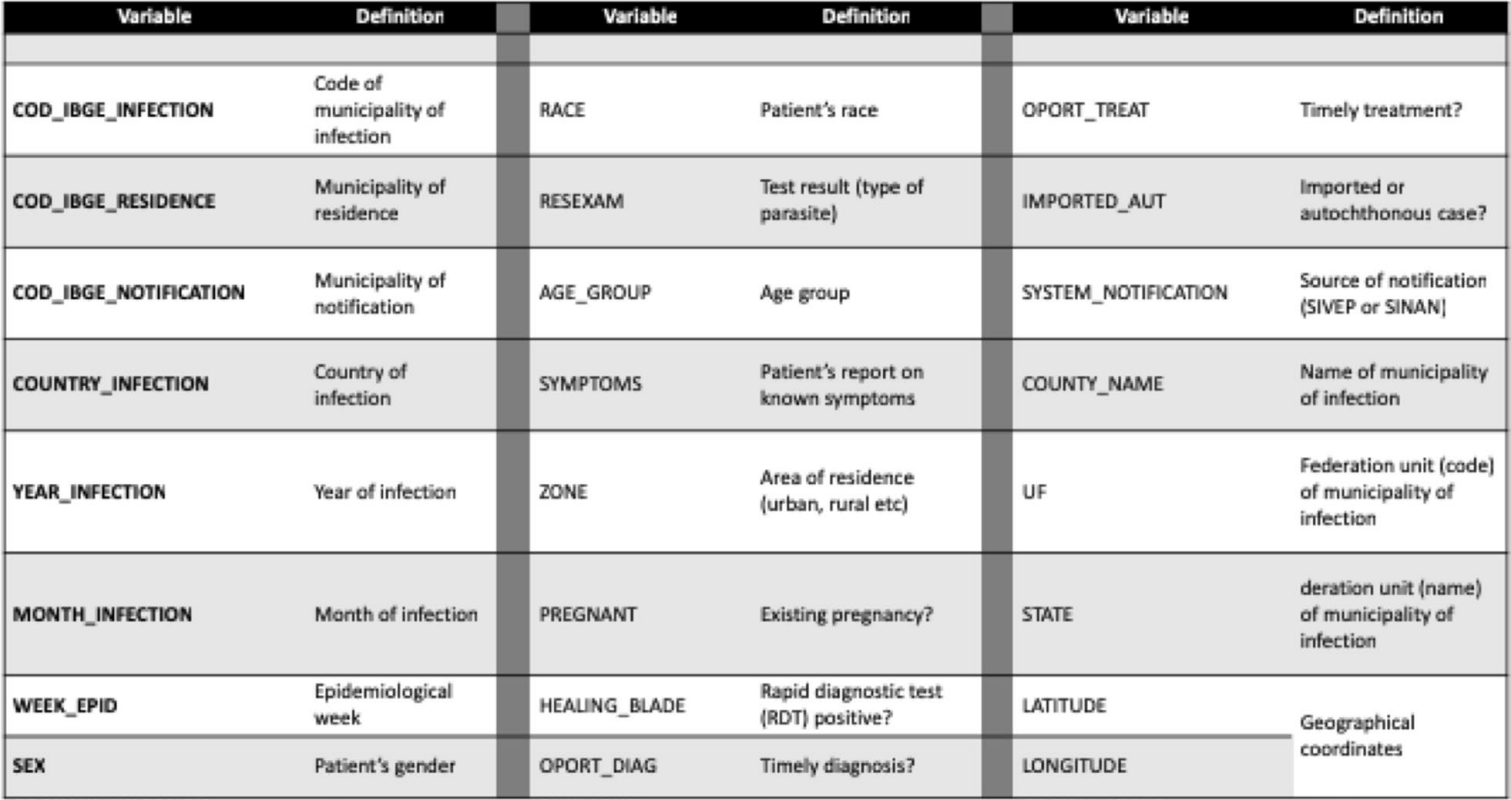
Structure of the national database of malaria episodes

The data linkage flow is depicted in Fig. 2, while Fig. 3 summarizes the main attributes aggregated into the “national database of malaria episodes”. This database has a mixture of raw data (variables from SIVEP and SINAN), as well new variables storing information about timely or late diagnosis and treatment, imported and autochthonous cases, epidemiological week, and geographic coordinates.

### Data cleansing and harmonization

Data cleansing is a process used to improve data quality through the correction of detected errors and omissions. Correcting errors in data and eliminating bad records can be a time consuming and tedious process [12], but it cannot be ignored. It is important and required when integrated data from several databases, since the structure of each database differs from the others.

In this work, all databases were analysed for error and data missing patterns, and then passed through standardization routines to improve data quality and ensure accurate results when linked together. Most of the errors encountered were imputation errors, in attributes such as age, sex and race. In general, data cleansing and harmonization strategy consisted of (i) replacing missing data with a’999’ variable, (ii) standardization of dates, and (iii) aggregation of different attributes combining the results into a new variable.

### Visual data mining

Visual Data Mining is a subject that has been receiving prominence in the academic environment in the last years. Visualisation techniques are proposed to deal with two big aspects of visualisation: Visual Exploratory Analysis (one of the aspects of Information Visualisation) and Data Mining, that aims at to aid in the process of knowledge acquisition through graphical representations to explore and analyse large databases.

Visualisation has many definitions but the most referred one found in literature is “the use of computer-supported, interactive, visual representations of data to amplify cognition”, where cognition means the power of human perception or, in simple words, the acquisition or use of knowledge [13]. This work has explored how visualisation can support decision making and improve the Brazilian malaria surveillance ecosystem. By means of different representations that can expand human cognition, the goal was to map and provide understanding of data from different databases in a graphical format, based on visual representations and interactive mechanisms.

**Fig. 4:**
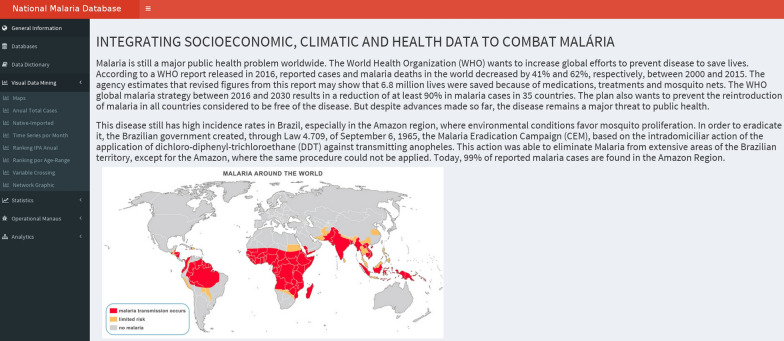
Malaria-VisAnalytics main interface

Besides building a national database of malaria episodes, this work has also concentrated on the design of Malaria-VisAnalytics, a graphical mining tool providing different features to allow for descriptive and predictive analyses over the integrated database. Figure 4 depicts the main interface.

## Results

In this section, different visualisation techniques capable of providing answers to data analysts and important considerations to discover new insights for decision-making are presented.

### Graph visualisation techniques

The visual data mining section of the Malaria-VisAnalytics platform allows researchers to visualize malaria cases based on the use of healing checking blades (LVC in Portuguese) or not. LVCs are important indicators for detecting deficiencies in health services related to the monitoring of sources of infection, care, and treatment of patients with malaria.

**Fig. 5:**
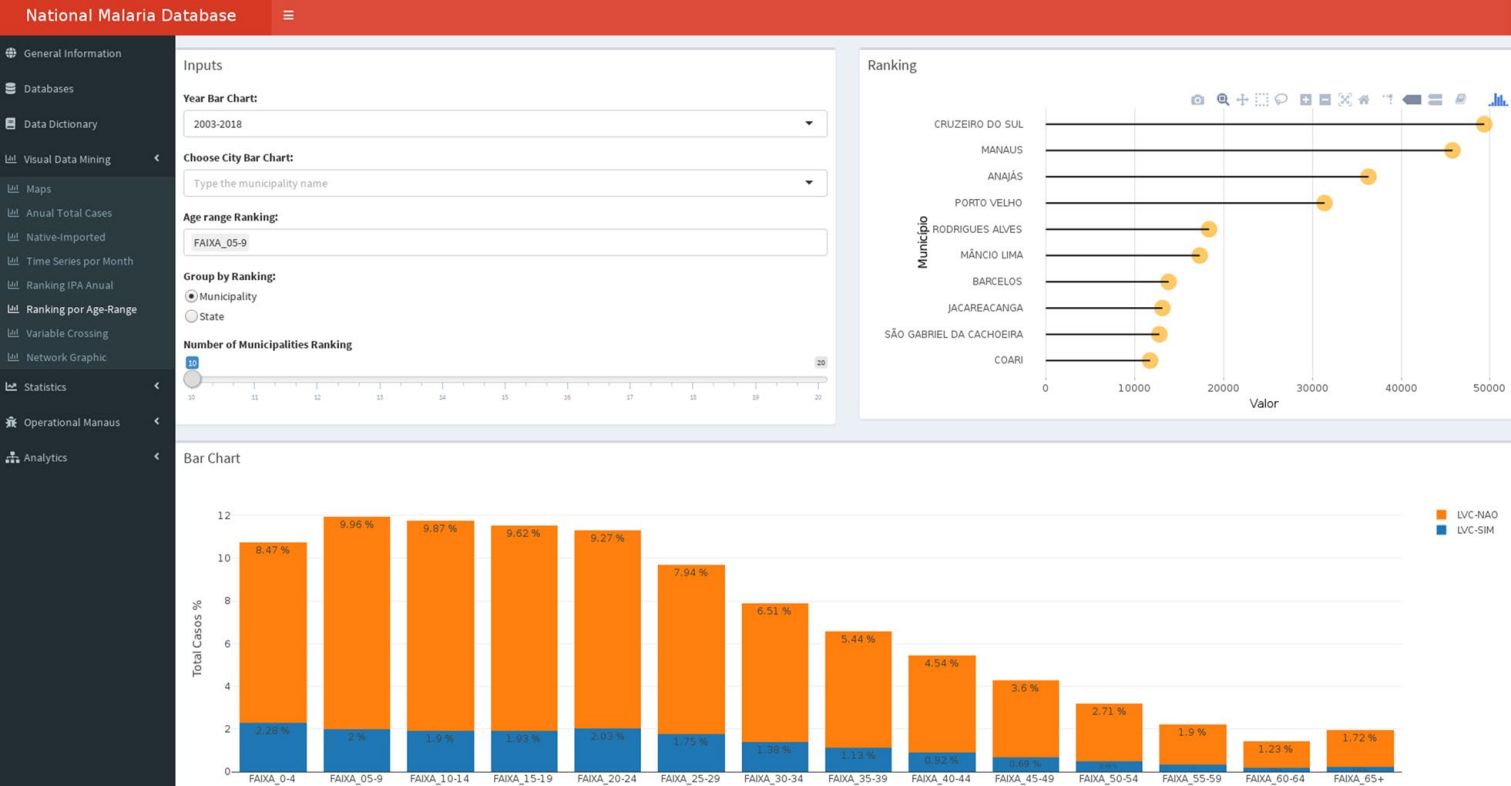
Example visualisation: number of infections by age range (“FAIXA”) and municipality

In Fig. 5, stacked bars were used to represent distributions along a one-dimensional ordered scale [14]. It is important to note that the highest recurrence of malaria is related to people under 30 years of age (“FAIXA 0–4” to “FAIXA 25–29”). Another important statistic (top right graph) shows the classification of the municipality by age group, which is important to analyse which city was most affected by the recurrence of malaria cases.

**Fig. 6:**
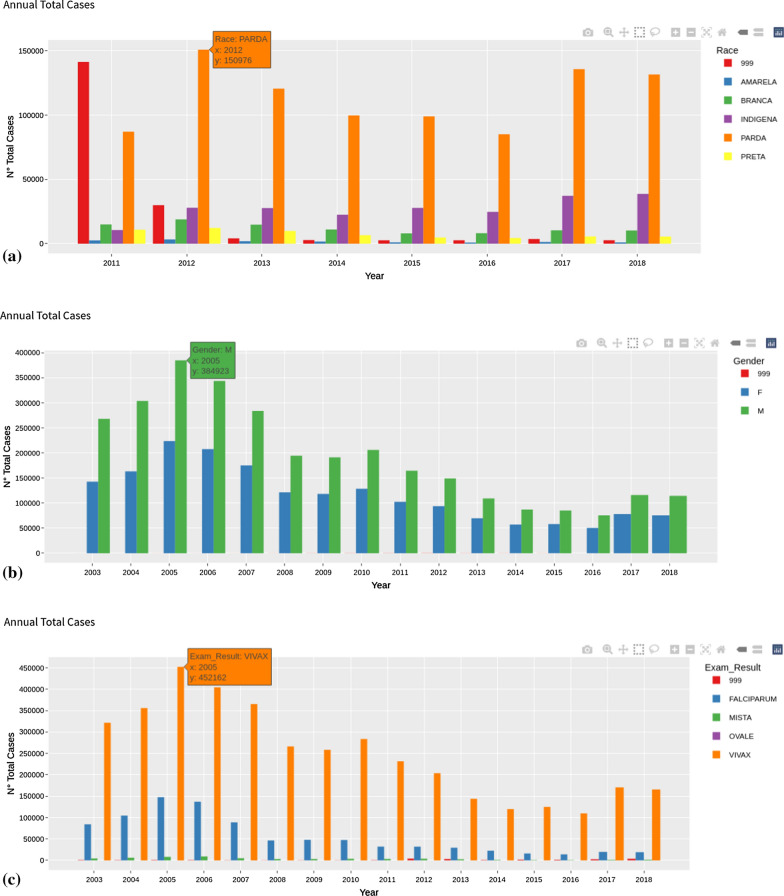
Total number of cases by race (**a**), gender (**b**) and type of *Plasmodium* (**c**). Null values are recorded as 999 in the database

To understand the trend of some basic variables, a bar graph was used, where the *X* axis represents the years and the *Y axis* represents their associated frequencies. Through these simple graphs, it is possible to identify which population is most affected by malaria (female or male), which race and the prevalence of infection. This type of chart is particularly suitable for qualitative or quantitative analysis of variables, since it highlights the presence of trends in the data, as shown in Fig. 6, respectively for race (a), gender (b), and type of *Plasmodium* (c).

**Fig. 7:**
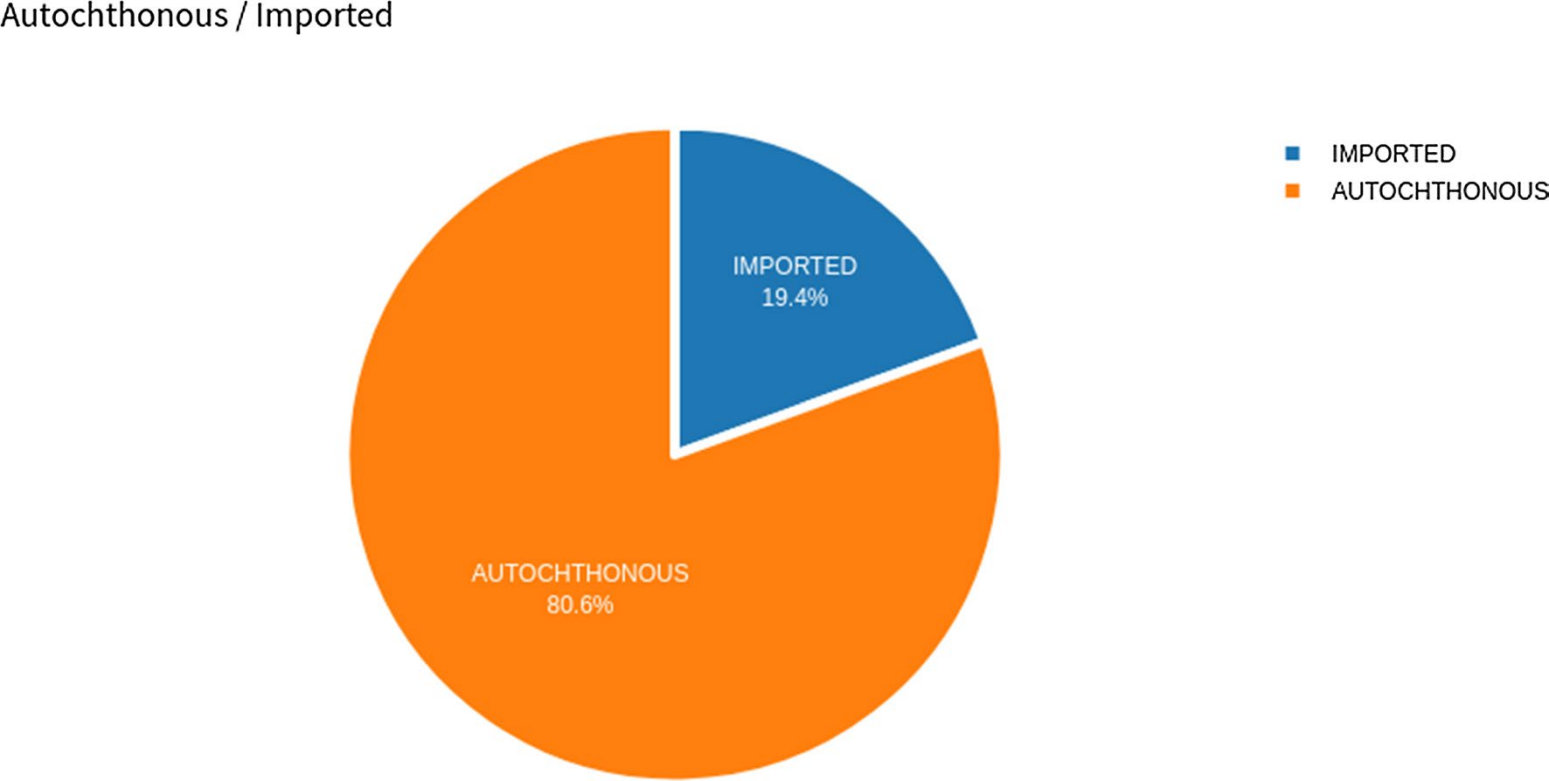
Autochthonous versus imported cases in all country from 2011 to 2018

Factors related to imported malaria cases are a key determinant of autochthonous malaria transmission, and may be the missing piece to understand how malaria patterns might change in the future. Examining the potential for autochthonous malaria transmission inside an area of previous outbreaks allows for a better understanding of the key populations facilitating or hindering transmission: the mosquito vector and the human host. Figure 7 shows autochthonous and imported cases based on the aggregated data. *Autochthonous malaria* represents cases where the disease was acquired in the same residential area as the patient, whereas *imported cases* relate to transmission occurring outside the patient’s residential area. This is an important metric for surveillance and combat actions during outbreaks.

**Fig. 8:**
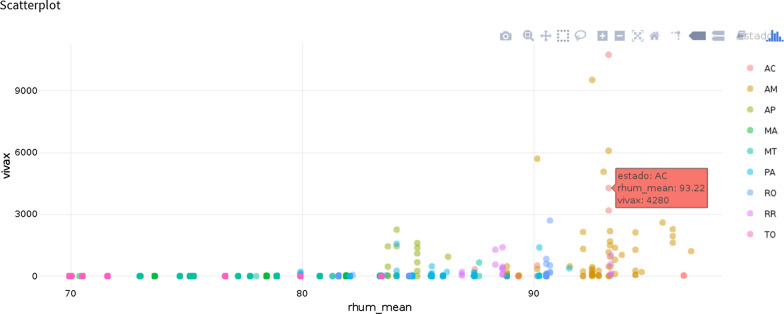
Example of bivariate analysis of infection by *Plasmodium vivax* versus humidity (*rhum_mean*) for different federation units

Climate conditions, especially precipitation and temperature, are important factors affecting the life cycle and longevity of malaria vectors. This results from the seasonality of malaria in several regions of the planet. The emergence of climate change because of global warming, popularized as a “greenhouse effect”, has been a subject for conjecture, because it has the potential to influence the increase of Anopheline populations that transmit malaria throughout the tropical zone [15]. Due to this consideration, some important variables were analysed through scatter plots, as they are a powerful graphic metaphor to inspect relationships between pairs of quantitative variables [16]. By exploring the relationship between those two variables, climatic and total number of cases per species, as shown in Fig. 8, some important patterns were observed due to the usefulness of multivariate scatter plots for such types of exploration.

**Fig. 9:**
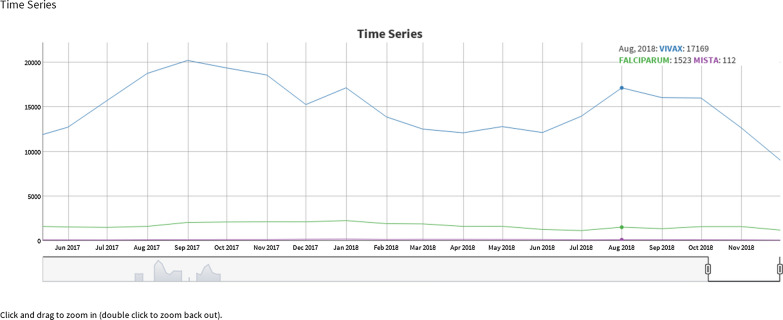
Time series visualisation of the evolution of malaria cases by type of *Plasmodium*

**Fig. 10:**
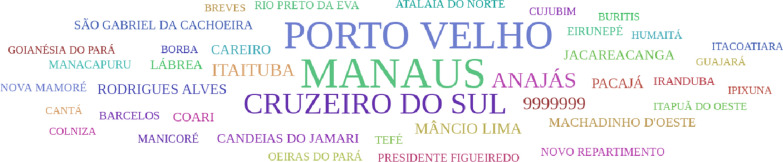
Word cloud of most affected cities

According to [17], “time series are sets of measures of the same magnitude, relating to several consecutive periods”. That is, a time series is a succession of values of a given variable observed at regular intervals of time. The control variable is time and varying the order can modify the information contained in the series [18]. They can be collected at regular intervals of time and analysed daily, monthly, quarterly, annually among other options. As an example, Fig. 9 shows the distribution of different species of malaria parasites, which refers to information on the evolutionary behaviour of the disease over the years. A cloud of words is mainly used to identify the most used words in a text, to know the concepts emphasized and to analyse the density of these identified keywords. It provides an attractive graphic element that presents a characteristic that distinguishes it from the others. The text does not have to be structured and it is possible to understand the information with a simple overview [19]. In this context, the idea of the Animated Word Cloud shown in Fig. 10 is to present information about which city is most affected from malaria. The technique uses the differences in text size to highlight the quantity of malaria cases in each municipality.

**Fig. 11:**
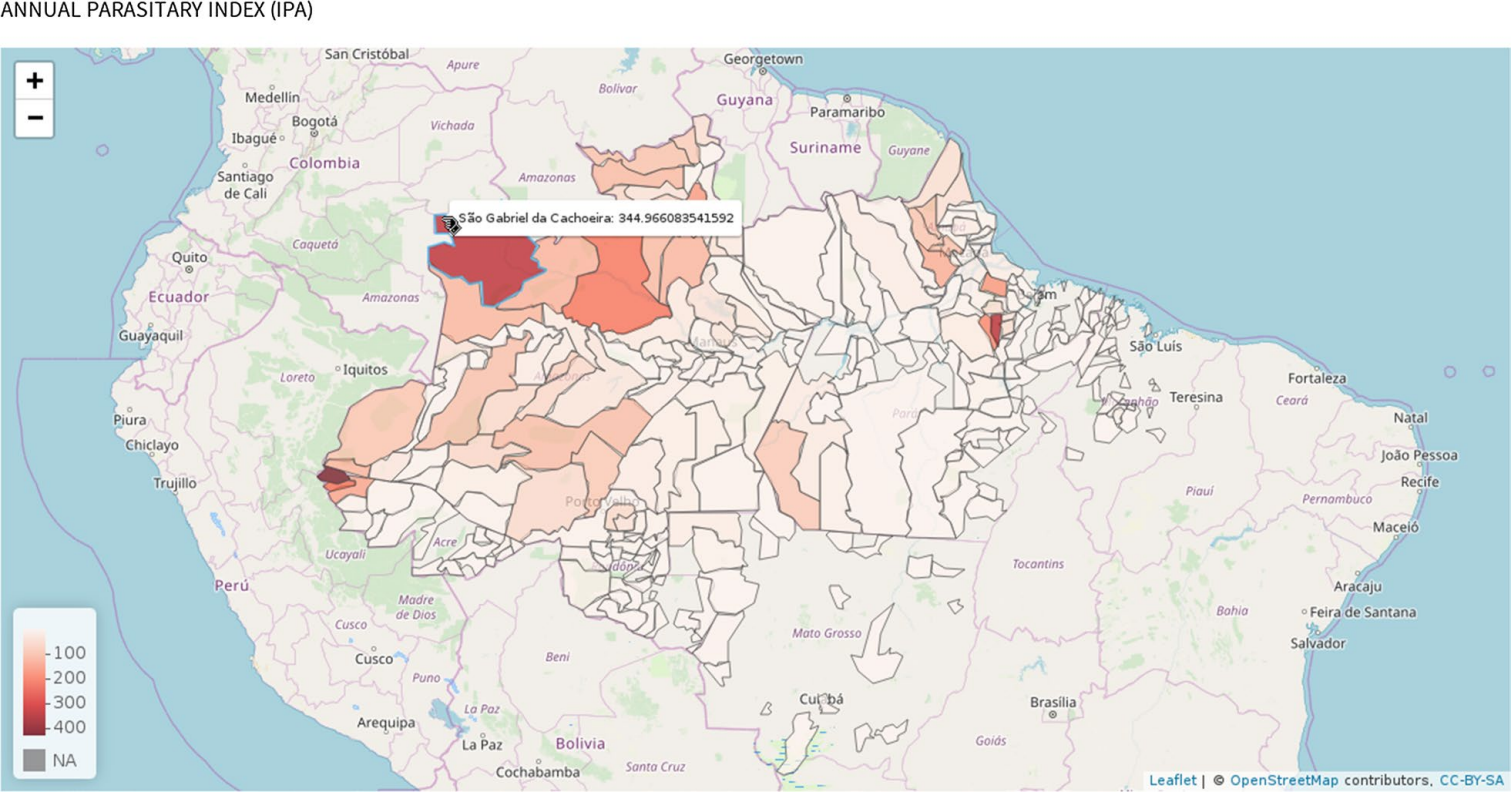
Choropleth map of malaria incidence inside the Amazonian region

The potential of geo-localization technologies becomes more evident considering the emergence of online interfaces based on geographic mapping [20]. Choropleth maps allow the visualisation of the spatial distribution of malaria incidence rates, as depicted in Fig. 11 with emphasis on the Amazonian region. The variation of value (tone) in each municipality indicates the incidence of malaria. Darker shades are equivalent to regions with a higher concentration of malaria cases, while lighter shades indicate regions with lower incidence of the disease.

Interactive visualisation and exploration of the graph structure and connectivity patterns (e.g., nodes and edges) can answer a lot of research questions. One of those questions can be the number of *k*-core connections for each area under study [5], as this metric allows for identifying most cohesive areas (biggest k-core values) which represent neighbouring cities with high degrees of disease transmission.

**Fig. 12:**
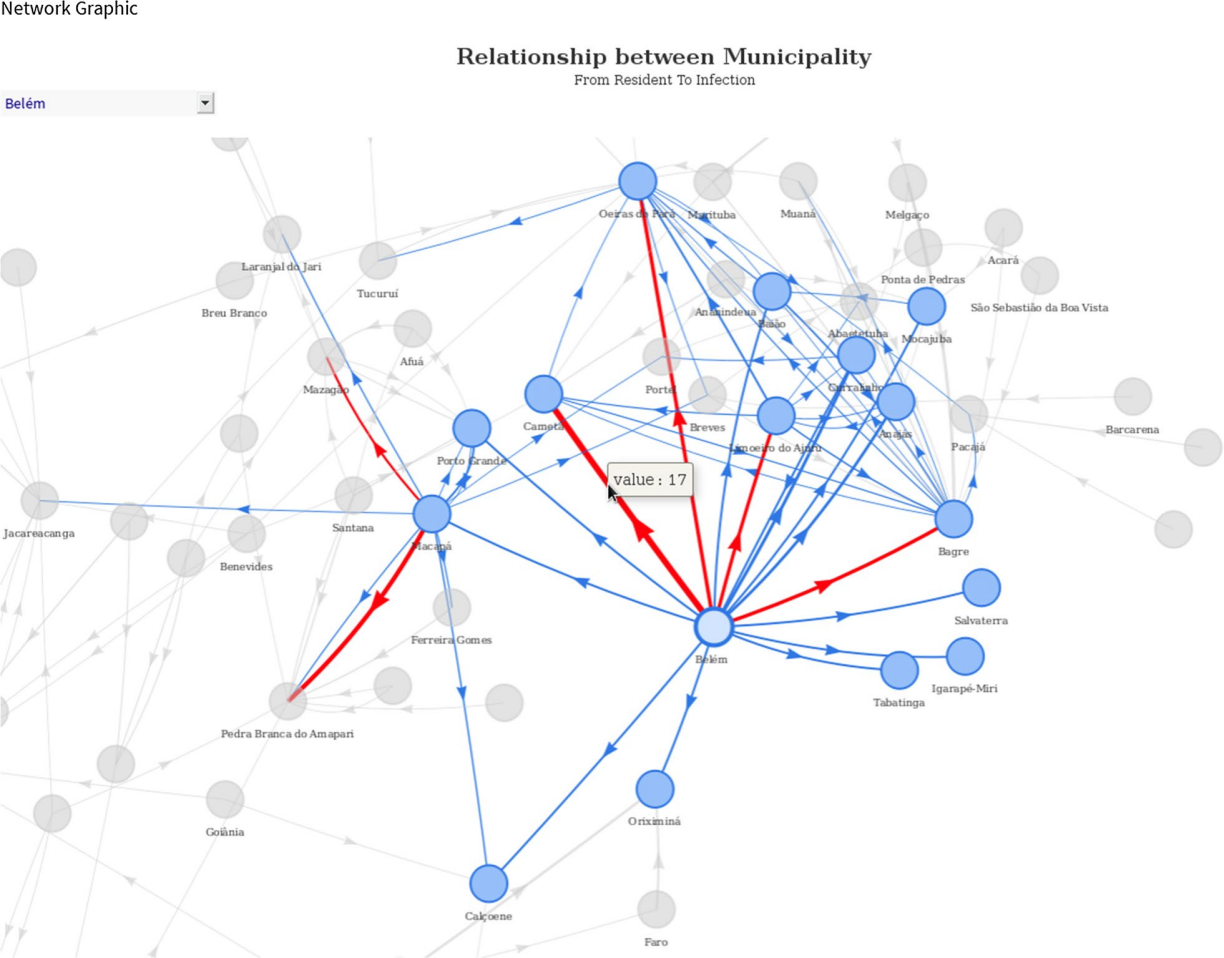
Graph network of infections contracted outside Belém by its residents (2018 extract). Values in the red lines indicate how many people were infected outside their residential area

Through a graph network structure, it is possible to discover valuable insights in a more intuitive way that other types of visualisations are not able to provide. Figure 12 shows a network graph to find interactions among municipality of residency and municipality of infection. After applying some filtering and visualisation patterns, it was noticed that most people who live in Belém, for example, contracted an infection outside their home. Only the edges where interactions have more than five people were highlighted in red, referring to the year 2018.

### Integrating spatial and non-spatial visualisation

To know more about the health conditions of a given population, it is necessary to work with maps that allow for the observation of spatial distribution of risk situations and health problems, with demographic, and environmental data, promoting the integration of different information databases. In this sense, it is fundamental that the information be georeferenced, providing elements for building the chain of explanations of problems from a given territory and increasing the power to guide specific actions [21].

#### Case study: Manaus

**Fig. 13:**
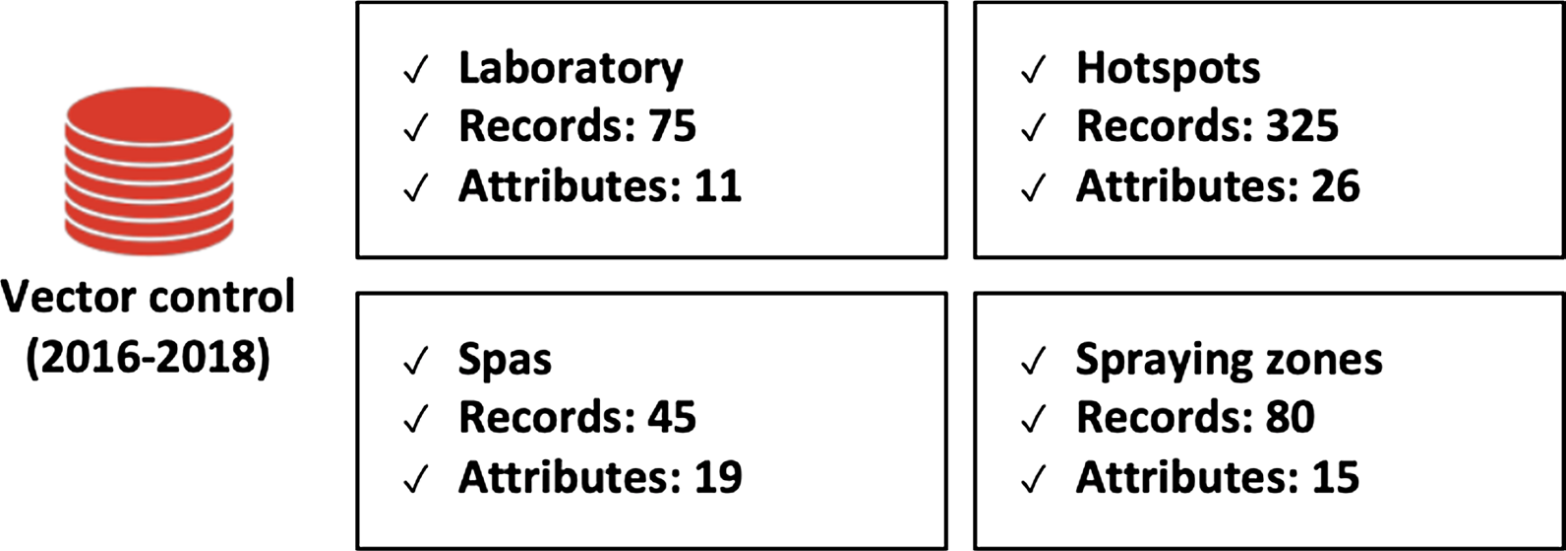
Database of vector control data used in the Manaus case study: laboratory, hotspots, spraying zones, and spas/balneary regions

Spatial and non-spatial data were integrated for a case study in Manaus, capital of Amazonas, the state that consistently reports the highest number of malaria cases in Brazil. For this case study, data on transmission vectors, laboratory data, breeding sites and water/spa zones were also aggregated, as illustrated in Fig. 13. Mixed geospatial and non-spatial visualisation techniques were combined into a multi-layered system to provide timely information and allow for better management of resources to fight malaria at the municipal level.

To be mapped, each data item must be referenced to a geographic analysis unit. Most of the Brazilian health databases have a municipality code used for geo-localization purposes. On the other hand, the cartographic bases must contain fields that allow the relationship with databases. It is from the relationship between the database and the cartographic base that one can perform several common geoprocessing procedures, such as geostatistical analysis, graphical and non-graphical information management, spatial operations, and graphical representation of results [22].

**Fig. 14:**
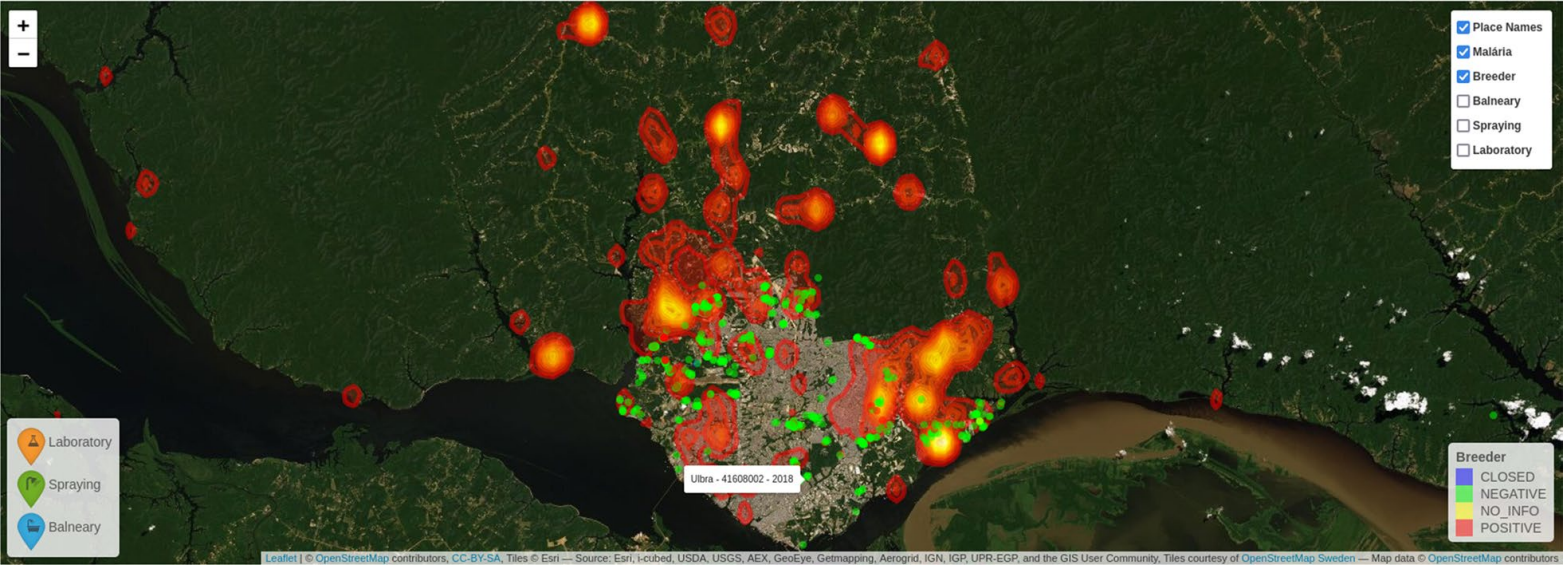
Correlation analysis of hotspots and reported infection areas

**Fig. 15:**
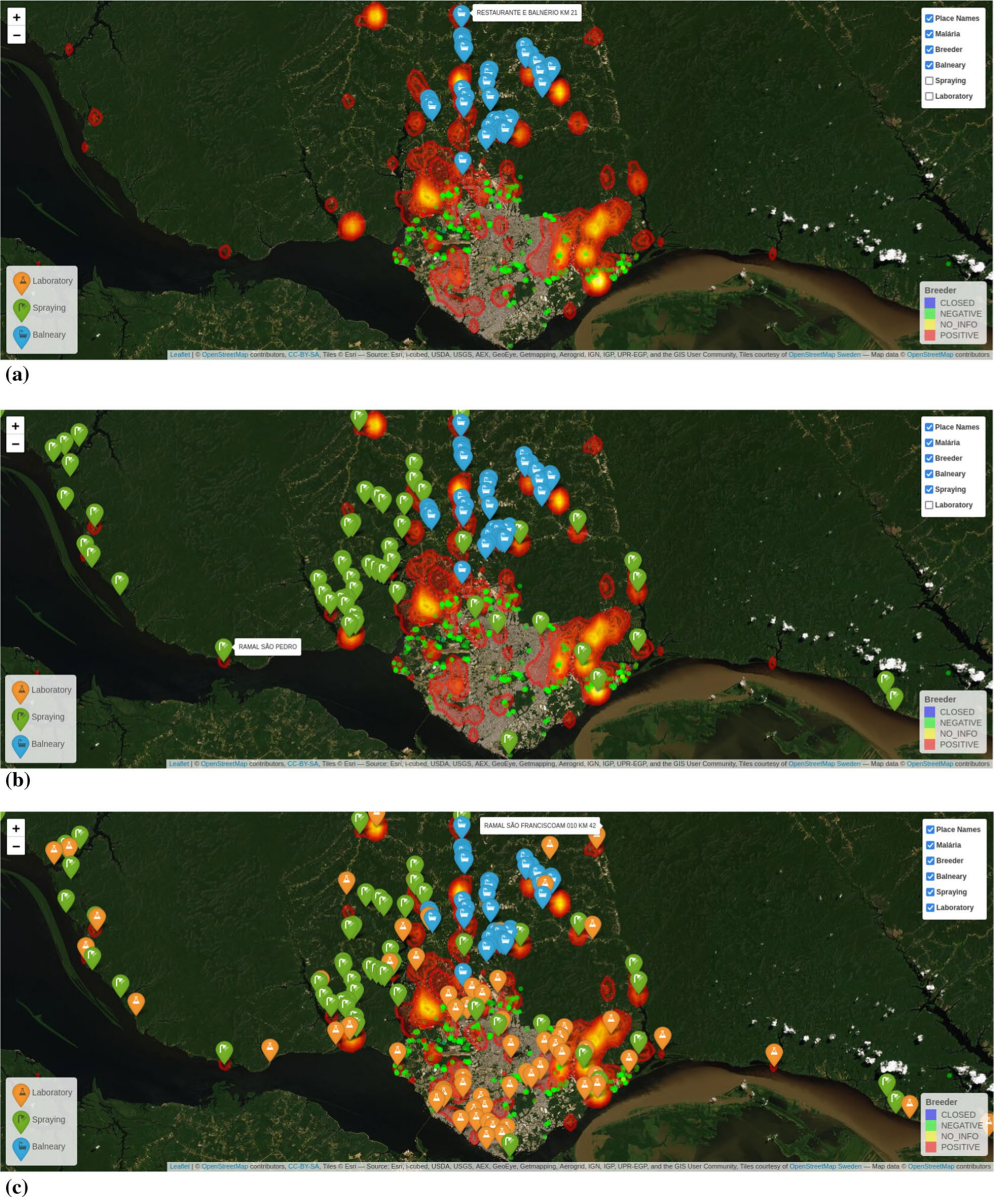
Multi-layered visualisation of Manaus showing spas/balneary (**a**), spraying zones (**b**) and laboratories (**c**), along with hotspots/breeders. Reported infection areas are shown in yellow/orange

The points in Fig. 14 indicate categorization of different hot spots (breeding sites) in the city (negative, positive, closed, no info). The comparison with the heat-map is important to verify the relationship between malaria cases and hot spots. Figure 15 shows additional layers of information (spa/balneary spots, spraying zones and laboratories) that can be overlapped for better decision-making related to malaria in Manaus. This information is important to support real time control during outbreaks for controlling malaria spread, checking whether a given spot was sprayed out, and determining which part of the city needs additional laboratory facilities, respectively. Several risk areas can be delimited, and faster decision can be made using associations between layers (maps) of information.

### Interaction techniques

Interaction techniques are necessary for an effective data exploration instead of using only visualisation techniques. They allow data analysts to change the visualisation according to exploration needs, directly interacting with the visualisation and not only seeing the data. In the proposed tool, multiple independent visualisations can be combined and related through different interaction techniques. Navigation techniques are powerful and can easily modify the projection of the data on the display to better understand the data and have more answers [23].

#### Interactive filtering

**Fig. 16:**
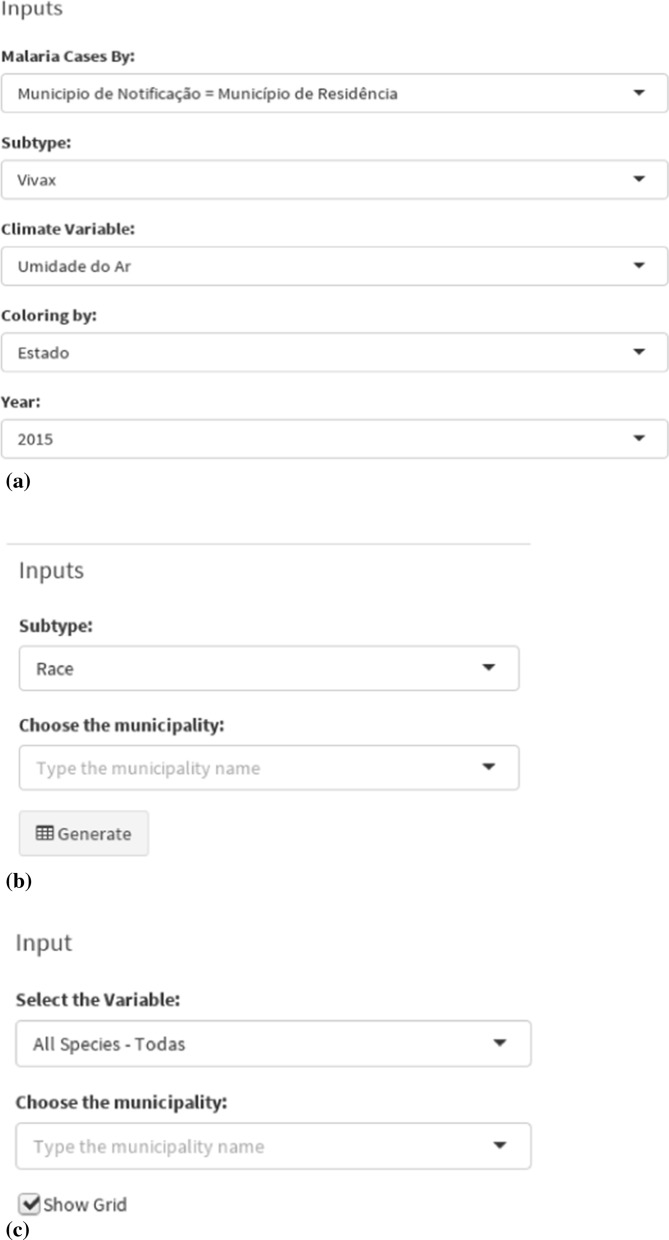
Example of interactive filters provided by the Malaria-VisAnalytics tool: (**a**) Inputs by type of cases, subtype of *Plasmodium*, climate variable, year, and highlights (coloring) by state or region; (**b**) Inputs by subtype (race or gender) and municipalities; and (**c**) Inputs by species and municipalities

When dealing with the exploration of large data sets, it is important to focus on interesting subsets of data those final users want to visualize and interactively partition the data into different segments. Specialist support was sought on how to implement interactive filtering based on the needs of intended final users. This process went through the selection of the desired subsection (browsing) and a custom specification of the properties according to the subset of data (querying). Another important point evaluated was data volume: when the data set is very large, browsing is very difficult due to the real time analysis, and querying do not produce the desired results. This can be solved through code optimization and support of good computational power. Therefore, in the developed tool, interactive filtering and visualization have been improved thanks to the development of interactive selection techniques [24], as depicted in Fig. 16.

#### Zooming

During the process of detecting new patterns through visualisation, it is important to consider view modification techniques (or zooming) that are widely used in several applications. In the scenario of this work, it was necessary to deal with a large amount of data through different visualisation approaches, so it is important to allow a variable display of the data at different resolutions. When zooming the map, it is necessary not only increase the size of the data object on the display, but also automatically change the representation of the data to present more detail with higher zoom levels [24].

**Fig. 17:**
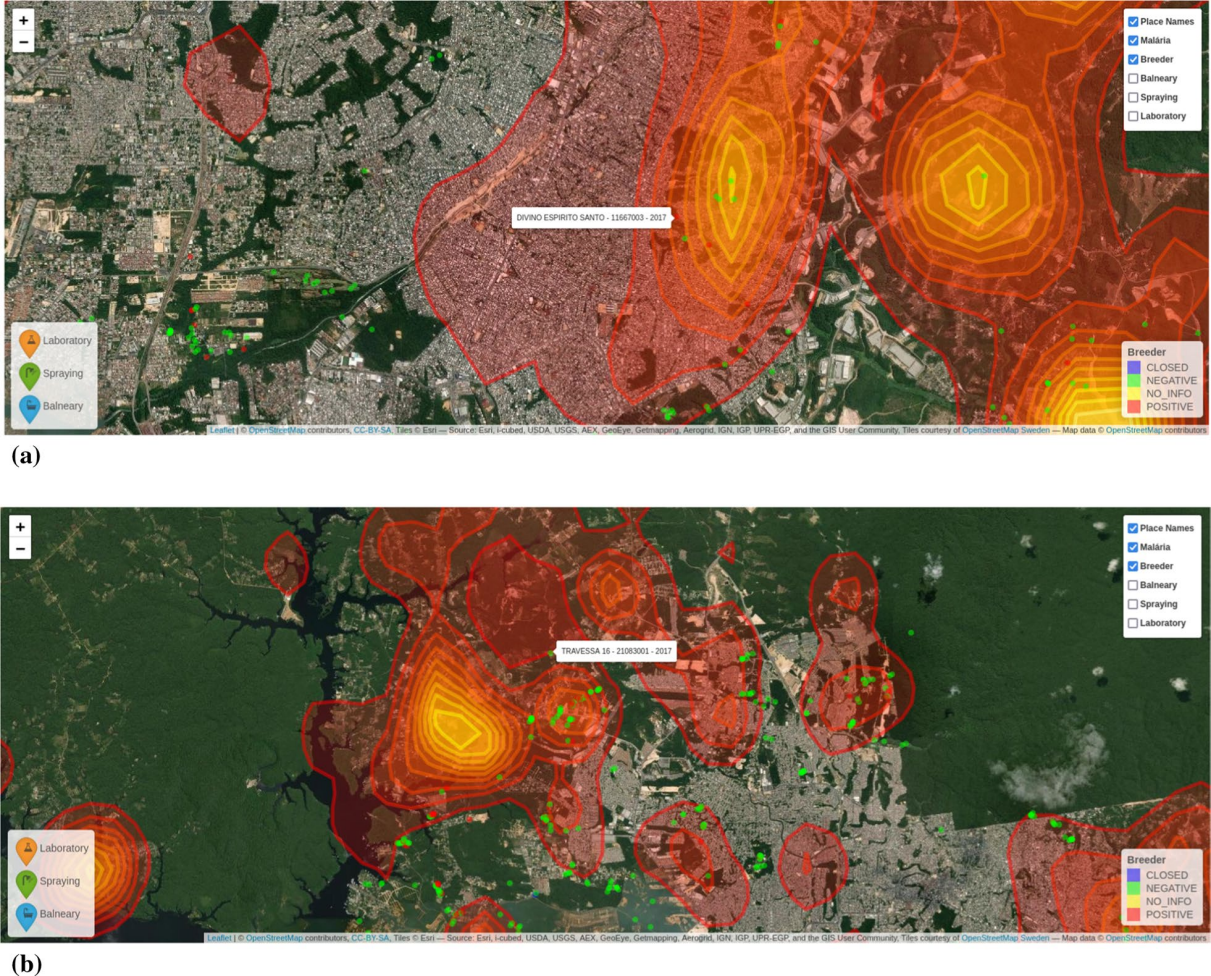
Example of zooming techniques applied over Manaus, at different scales (**a** and **b**)

For a better understanding of the zooming techniques shown in Fig. 17, it is possible to note that mosquito hot spots are in the area where malaria cases are most concentrated. Then, thanks to the zoom techniques applied at different scales, it is possible to see that some hot spots not yet treated are causing the contamination and, in this case, make an informed decision about necessary actions to prevent the spread of the disease.

## Discussion

Malaria-VisAnalytics (MVA) allows users to visualize and analyse static data through an user-friendly Web interface. MVA gives users the possibility to interactively explore and compare data along many different techniques and dimensions. The goal of MVA is to make it easy for users to quickly discover key insights into the data with little effort, while also providing a medium for researchers to share data, visualisations, and insights, ask problem-specific questions and make the decision process faster and more effective. Health professionals can benefit from different visual resources, ranging from a more global chlorophetic visualisation allowing for analysing how a given variable change from one region to another; to more specific visualisations, such as a network graph, which shows the relationship between multiple nodes (in this case, municipalities).

Data visualisation can help users to draw actionable insights from massive amounts of data in a short amount of time. Even a simple visualisation, like a bar graph, can present valuable insights in seconds. Data visualisation also simplifies the information, like in a comparative bar chart. Pie charts are ideal for illustrating proportions, or part-to-whole comparisons: in this specific study, it was used for comparing imported and autochthones cases.

Timelines allow the user to highlight the most important events that occurred, or are likely to occur in the future, and make easier for the viewer to identify any patterns appearing within the selected period; for instance, monitoring infections or hospitalization by malaria over the years. Scatter plots display data for two variables, represented by points plotted against the horizontal and vertical axes. This type of data visualisation is useful for illustrating existing relationships between variables and can be used to identify trends or correlations in data.

The proposed tool makes use of all these data visualisation resources for providing a comprehensive view of malaria episodes and attached variables contributing to the occurrence of such cases, as well as those variables that can contribute significantly to the understanding of different processes involved in surveillance and decision-making.

## Limitations

The contributions of the proposed tool must be seen considering some limitations. The first limitation refers to the periods that can be effectively tested, which are limited by the duration and sampling frequency of the input data. The input data are not easily accessible—data was granted upon request by the sanitary surveillance foundation and limited to the years of study. The data has also the limitation of not being easy to update, so hindering real-time analysis, which would be quite valuable and necessary for fast decision-making and for improving the control of malaria cases in specific regions.

Another limitation that the visualisation platform presents is related to the visual metaphors that are separated into different sections and not on a single screen, making comparisons between different visualisations impossible, which can lead the audience to a biased analysis. Another problem is that, although the developed application is explanatory, its clarity in explanation and interpretation completely depend on the focus of each particular audience: if the audience is not focusing on the central message for which the visualisation is presented, it may not seem satisfactory and, then remain unimportant in the central purpose of doing any analysis.

Regarding the case study on Manaus, the intention was to implement an automatic real-time vector control system to help reduce malaria cases in the region. Several limitations were encountered during the data collection phase: different systems hosted and managed by diverse health foundations made accurate visual analysis difficult and hinder the acquisition of up-to-date data.

There is no automated data collection system capable of storing the information in a single digitized system. The collection of data from laboratories, spas, and spraying zones is done exclusively manually, hindering a more accurate and timely control of the disease. Despite these limitations, Malaria-VisAnalytics could be implemented in other states, streamlining and helping the process of data collection and decision-making.

## Conclusions

With this work, the use of exploratory analysis and traditional visualisation tools that can enable the evaluation of high-dimensional data was illustrated. Malaria-VisAnalytics is a tool aimed to help researchers and health agents, especially those working on surveillance teams, to gain understanding of malaria episodes within and outside the Amazonian region, including other factors (such as climate data) directly or indirectly related to them. Future work could comprise fast data capture and real-time updates as soon as data are entered in any relevant database inside the malaria surveillance system. This would allow to keep the integrated database up-to-date and hence more realistic monitoring leading to more accurate decision-making. Potential contributions related to data sharing and global information dissemination can be also sought. Finally, the structure and design approach behind the proposed platform can serve as a benchmark for monitoring other diseases through visual data mining.

## Data Availability

The application is publicly available at http://ipef2020.ddns.net:3838/malaria/. Metadata and datasets can be downloaded from Sironi, Alberto; Junior, Juracy; Sampaio, Vanderson; Coimbra, Danilo; Rasella, Davide; Barreto, Marcos (2022), “MalariaVisAnalytics”, Mendeley Data, V1, https://doi.org/10.17632/d6426h6fhn.1.
